# Evaluation of eGFRs to Calculate Kidney Function in Adults with Severe Motor and Intellectual Disabilities

**DOI:** 10.31662/jmaj.2023-0069

**Published:** 2023-10-02

**Authors:** Osamu Uemura, Yuka Hasegawa, Hideaki Nakashima, Yoshihiro Otobe

**Affiliations:** 1 Department of Pediatrics, Ichinomiya Medical Treatment & Habilitation Center, Ichinomiya, Japan; 2 Department of Pharmacy, Ichinomiya Medical Treatment & Habilitation Center, Ichinomiya, Japan; 3 Department of Rehabilitation, Ichinomiya Medical Treatment & Habilitation Center, Ichinomiya, Japan

**Keywords:** validation, estimated glomerular filtration rate, severe motor and intellectual disabilities, creatinine, cystatin C

## Abstract

**Introduction:**

The gold standard for evaluating kidney function is kidney inulin clearance (Cin). However, this procedure is difficult to perform in patients with neuromuscular disabilities and/or bladder dysfunction. We aimed to develop a simpler method for determining the estimated glomerular filtration rate (eGFR) using equations and values for three biomarkers: serum creatinine (sCr), serum cystatin C (cysC), and serum beta-2 microglobulin (β2MG). This study evaluated three eGFR equations in patients with severe motor and intellectual disabilities (SMID).

**Methods:**

We evaluated the equations using data of 18 adult SMID patients with a clinical need for creatinine clearance (Ccr). We compared the results of each equation with Ccr-based eGFR instead of Cin using mean error (ME), root mean square error (RMSE), and P30.

**Results:**

Based on eGFR, the ME values of Cr, cysC, β2MG, and Ccr were 74.5, 2.3, and 6.5 mL/min/1.73 m^2^, RMSE values, 92.3, 25.7, and 33.4 mL/min/1.73 m^2^; and P30, 16.7%, 77.8%, and 72.2%, respectively.

**Conclusions:**

eGFR-Cr cannot be used to reliably assess kidney function in adult SMID patients. It is better to use eGFR-cysC to evaluate kidney function in this patient population.

## Introduction

Estimated glomerular filtration rate (eGFR) formulas using serum components such as creatinine are frequently used in children ^(1), (2), (3)^ and adults ^(4), (5)^. However, eGFR calculation in patients with severe motor and intellectual disabilities (SMID) using serum creatinine (sCr) overestimates kidney function due to low muscle mass in these patients ^(6)^. Elevated creatinine-based eGFR has been reported to be associated with sarcopenia, dysphagia, and adverse rehabilitation outcomes after stroke ^(7)^. In Japan, SMID patients are bedridden or only able to sit, crawl, or walk with support; furthermore, they have profound intellectual disability (IQ < 35) ^(8)^. This study aimed to evaluate and compare the efficacy of eGFR formulas in SMID patients. Cin is the gold standard for evaluating kidney function, but eGFR using creatinine clearance (Ccr) ^(4)^ is a simpler method and reduces patient burden. We evaluated and compared the efficacy of three eGFR equations in 18 SMID patients who underwent Ccr for clinical reasons.

## Materials and Methods

### Study population

Only SMID patients are admitted to our facility. Data of 18 SMID patients (10 men, 8 women; age range, 20-72 years) with or without chronic kidney disease (CKD) who underwent 24-h Ccr for clinical reasons between September 2021 and June 2023 were examined. In one patient, data was obtained from a retrial due to inadequate urine collection. Retrials are conducted if the daily Cr urinary excretion / (1.73/body surface area) is <30%. This figure is the ratio of a patient’s daily urinary Cr excretion to that of a healthy adult with the same body surface area, assuming that an adult with 1.73 m^2^ body surface area has a daily urinary Cr excretion of 1.0 g. In general, muscle mass in SMID patients was considered to be about 50% of that in healthy adults with the same body surface area.

### Method of Ccr and Ccr-based eGFR measurements

Ccr-based eGFR (eGFR-Ccr) was obtained from Ccr performed using the 24-h urine collection method. A formula developed to predict inulin clearance (Cin) from 24-h Ccr in Japanese adults was used, and the data was designated as eGFR-Ccr as described ^(4)^: eGFR-Ccr (mL/min/1.73 m^2^) = 0.715 × Ccr. Generally, Cin is the gold standard for evaluating kidney function; however, owing to its complexity, 24-h Ccr was used instead.

### eGFR calculations

The three eGFR formulas used to assess kidney function in SMID patients were Cr-based eGFR equation^(4)^, eGFR-Cr (mL/min/1.73 m^2^) = 194 × Cr^(−1.094)^ × age^(−0.287)^ × 0739 (if woman); serum cystatin C (cysC)-based eGFR equation ^(5)^, eGFR-cysC (mL/min/1.73 m^2^) ＝ 104 × cysC^(−1.019)^ × 0.996^(age)^ × 0.929 (if woman) − 8; and serum beta-2 microglobulin (β2MG)-based eGFR equation ^(3)^, eGFR-β2MG (mL/min/1.73 m^2^) = 149.0 × 1/β2MG + 9.156. Because there is no β2MG-based eGFR formula for adults, we used one for children.

### Statistical analyses

To compare eGFR-Cr, eGFR-cysC, and eGFR-β2MG with eGFR-Ccr, we used three methods:

1) Mean error (ME): the difference between each value for eGFR and eGFR-Ccr

2) Root mean square error (RMSE): the square root of the mean square error for each eGFR and the eGFR-Ccr value

3) P30: the percentage of each eGFR value within 30% of the eGFR-Ccr value.

These three methods were used to assess the reliability and validity of the three obtained eGFR.

All statistical analyses were conducted using SPSS Statistics 26 (IBM Corporation) and Microsoft Excel 2016.

### Additional research

Data of 111 SMID patients (56 men, 55 women; age range, 20-74 years) obtained in our facility between February 2018 and January 2023 were evaluated for distributions of kidney function, CKD stage classification, and muscle mass in the patient cohort. Muscle mass evaluated by the ratio of eGFR-cysC to eGFR-Cr was expressed as a percentage of the general population as previously described ^(9)^.

This study was approved by the ethics committee of Ichinomiya Medical Treatment & Habilitation Center (approval number: 2021037).

## Results

### Study population characteristics

Of 117 long-term-admission SMID patients in Ichinomiya Medical Treatment & Habilitation Center, data of 18 patients (10 men, 8 women; age range, 20-72 years) who underwent Ccr for clinical reasons were included (Table 1). Table 2 presents the laboratory data. Regarding their serum albumin levels, the median level was 3.9 mg/dL (minimum 3.1, maximum 4.5), and none were below 3.0.

**Table 1.** Demographic and Clinical Characteristics of 18 SMID Patients.

		Number of cases
Sex	Male	10
	Female	8
Age	20~39 years	9
	40~59 years	7
	60~79 years	2

Underlying disease	Cerebral palsy	10
	Congenital brain damage	3
	Brain malformation	2
	CNS infection sequelae	1
	Other	2

**Table 2.** Laboratory Data the 18 SMID Patients.

case	sex	age	height	weight	BSA	CCr	CCr-eGFR	Cr	Cr-eGFR	cysC	cysC-eGFR	β2MG	β2MG-eGFR	Alb
		(y)	(cm)	(kg)	(m^2^)	(ml/m/1.73m^2^)	(mg/dL)	(ml/m/1.73m^2^)	(mg/L)	(ml/m/1.73m^2^)	(mg/)	(ml/m/1.73m^2^)	(g/dL)
1	M	20	158.0	34.4	1.27	154.3	117.9	0.35	258.9	0.64	143.3	0.9	174.7	4.2
2	M	21	160.0	34.4	1.28	186.5	142.5	0.43	203.8	0.77	116.8	1.4	115.6	4.5
3	M	30	142.0	37.8	1.22	143.8	109.9	0.41	193.9	0.84	102.1	1.8	91.9	3.9
4	M	31	151.5	33.9	1.22	100.2	76.6	0.29	280.5	0.94	89.8	1.7	96.8	4.5
5	M	36	155.0	38.2	1.31	107.2	81.9	0.51	144.9	1.12	72.2	2.2	76.9	4.5
6	M	48	151.0	48.1	1.42	109.1	83.4	0.50	136.3	0.76	105.5	1.6	102.3	4.0
7	M	49	150.0	37.4	1.27	122.3	93.5	0.61	109.0	1.03	74.9	1.9	87.6	3.9
8	M	50	148.0	27.9	1.11	83.6	63.9	0.60	110.4	1.14	66.5	2.2	76.9	3.9
9	M	52	145.0	31.9	1.15	50.1	38.3	0.64	101.7	1.09	69.3	2.5	68.8	3.4
10	M	60	186.0	61.2	1.82	90.1	68.8	0.47	136.8	0.97	76.3	2.7	64.3	3.1
11	F	26	156.2	40.0	1.34	127.3	97.3	0.32	195.8	0.56	149.2	0.9	174.7	4.4
12	F	37	148.0	40.5	1.30	126.2	96.4	0.48	113.5	0.79	97.9	1.4	115.6	4.1
13	F	39	129.0	34.7	1.10	81.8	62.5	0.51	104.6	1.15	63.7	3.0	58.8	3.7
14	F	40	141.0	31.3	1.12	221.6	69.3	0.17	345.6	0.67	115.8	1.3	123.8	4.1
15	F	50	164.5	57.5	1.63	70.0	53.5	0.38	134.4	0.87	83.1	2.4	71.2	3.3
16	F	72	143.5	39.5	1.25	105.3	80.5	0.31	151.3	0.76	87.8	2.1	80.1	3.3
17	F	55	148.0	39.8	1.29	105.7	80.7	0.30	169.4	0.81	88.1	1.4	115.6	3.9
18	F	35	140.0	30.4	1.10	255.9	195.5	0.35	163.0	0.53	152.4	1.2	133.3	3.2

### Results of statistical analyses

**Figure 1.**
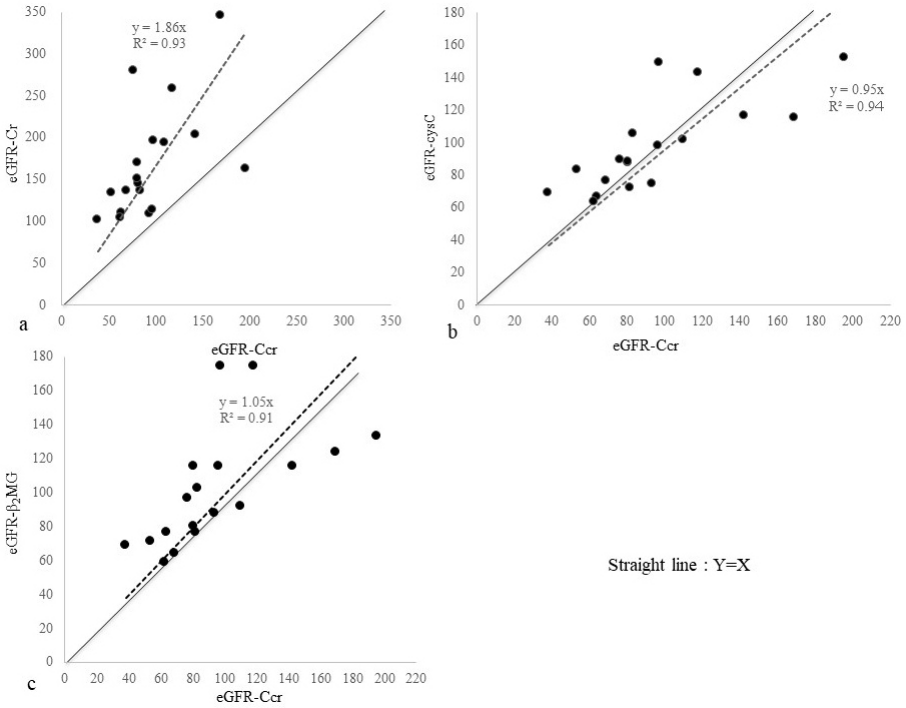
Scatterplot of eGFR-Ccr vs. 3 eGFR equations. a: eGFR-Cr b: eGFR-cysC c: eGFR-β2MG Straight line: Y = X Dashed line: Regression line passing through the origin.

Figure 1 presents a scatter diagram of eGFR-Ccr vs. eGFR equations. The straight line indicates equivalence among the eGFR-Ccr and eGFR values. The regression line passing through the origin is indicated by a dashed line, and the regression equation and coefficient of determination are shown.

**Figure 2.**
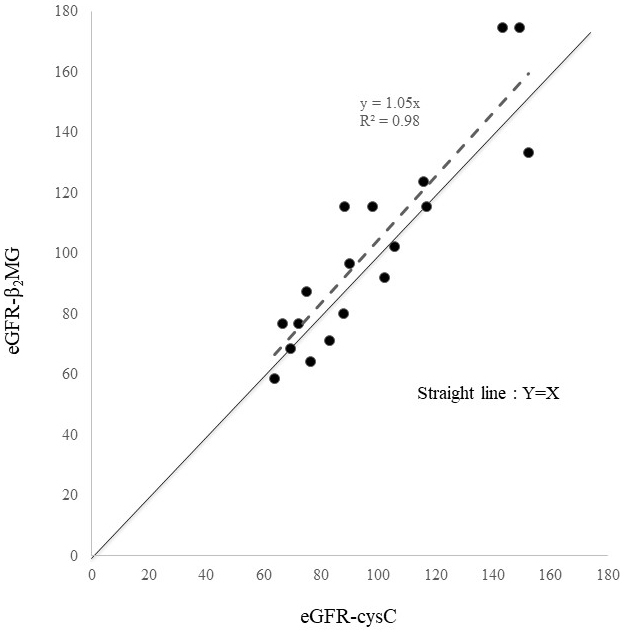
Scatterplot of eGFR-cysC vs. eGFR-β2MG. Straight line: Y = X Dashed line: Regression line passing through the origin.

We found eGFR-Cr to greatly overestimate kidney function in SMID patients. Contrarily, eGFR-cysC and eGFR-β2MG seemed to reasonably evaluate kidney function in these patients. Figure 2 presents a scatter diagram of eGFR-cysC vs. eGFR-β2MG. These two formulas showed considerable agreement, although eGFR-β2MG was a glomerular filtration rate (GFR) estimator designed for children.

Table 3 shows numerical data used to evaluate the reliability and validity of the three formulas. The ME values of eGFR-Cr, eGFR-cysC, and eGFR-β2MG were 74.5, 2.3, and 6.5 mL/min/1.73m^2^; RMSE values, 92.3, 25.7, and 33.4 mL/min/1.73m^2^; and P30, 16.7, 77.8, and 72.2%, respectively. Of all analyses, eGFR-cysC had the lowest ME and RMSE values and the highest P30.

**Table 3.** Comparison of Performance among the Three eGFR Equations, eGFR-Cr, eGFR-cysC, and eGFR-Β2MG, Using eGFR-Ccr as the Gold Standard.

	ME (mL/min/1.73 m^2^) (95% CI)	RMSE (mL/min/1.73 m^2^)	P30 (%)
eGFR-Cr	74.5 (−12.1 to 192.2)	92.3	16.7
eGFR-cysC	2.3 (−49.1 to 43.1)	25.7	77.8
eGFR-Β2MG	6.5 (−55.1 to 68.7)	33.4	72.2

*eGFR*: estimated glomerular filtration rate, *ME*: mean error, *RMSE*: root mean square error

### Kidney function and muscle mass in SMID patients in our facility

In 111 patients, the mean kidney function was 87.1 ± 27.1 mL/min/1.73 m^2^ calculated using eGFR-cysC. For reference, their eGFR-Cr was 158.0 ± 75.6 mL/min/1.73 m^2^. In terms of CKD classification according to kidney function, 47 cases were classified as G1, 50 as G2, 11 as G3, 1 as G4, and 2 as G5, and CKD occurred in 14 cases (12.6%). Their mean muscle mass was 61.0% ± 20.6% compared with healthy adults.

## Discussion

It was reported that eGFR-cysC estimations of kidney function performed better than creatinine-based ones in patients with primary neuromuscular disease ^(6)^. In Japan, elevated creatinine-based eGFR has been associated with sarcopenia, dysphagia, and adverse rehabilitation outcomes after stroke ^(7)^. Another study demonstrated that the creatinine-to-cystatin C ratios were lower in SMID patients due to their decreased muscle mass ^(9)^. In patients with cirrhosis, eGFR-cysC could estimate kidney function more accurately than eGFR-Cr ^(10)^. The present study aimed to evaluate eGFR formulas in SMID patients. We expected eGFR-cysC to be better than eGFR-Cr in assessing kidney function in these patients. We used eGFR-Ccr instead of Cin to evaluate kidney function as it is a simple method with low burden on patients.

In the data of 18 SMID patients who underwent Ccr for clinical reasons, we used ME, RMSE, and P30 to evaluate the reliability and validity of eGFR equations. We found eGFR-cysC to be the most accurate with the lowest ME and RMSE values and the highest P30 (Table 3). Figure 2 presents the reliability of eGFR-β2MG. Despite being a pediatric formula, eGFR-β2MG performed almost as well as eGFR-cysC. Cystatin C is recommended to be used for the estimation of GFR in patients with loss of muscle ^(10), (11), (12)^; similar observation was made in SMID patients in this study. Furthermore, this is the first study to measure the Ccr systematically in SMID patients. Compared with eGFR-Cr, eGFR-cysC and eGFR-β2MG performed better in assessing kidney function in SMID patients. There is no eGFR-β2MG for adults. Therefore, we applied eGFR-β2MG for children to adults, albeit for SMID patients, we were able to demonstrate similar usefulness to eGFR-cysC. We hope that eGFR-β2MG for adults will be developed. However, it should be noted that infectious, inflammatory, and malignant diseases affect eGFR-β2MG, and thyroid disease affects eGFR-cysC and eGFR-β2MG ^(13)^. Furthermore, kidney function in lean patients should be assessed by GFR without correction for normalized body surface area, particularly for the dose determination of drugs excreted by the kidney. Using eGFR-cysC, we evaluated the kidney function of 111 SMID patients, and the mean value was 87.1 ± 27.1 mL/min/1.73 m^2^; CKD with GFR < 60 mL/min/1.73 m^2^ occurred in 14 cases (12.6%), which were not high compared with that in the Japanese population ^(13)^. About 13.3-14.8 million Japanese people aged ≥20 years were estimated to have decreased kidney function with GFR < 60 mL/min/1.73 m^2^ ^(14)^. Another study in Japan reported a mean eGFR of 75.0 ± 16.2 mL/min/1.73 m^2^ ^(15)^.

The mean muscle mass in the 18 SMID patients was 61.1% ± 15.8% of that in the healthy population. It was reported that Asians have a skeletal muscle mass of 23.6 ± 5.3 kg and a body weight of 62.0 ±10.4 kg ^(16)^. Contrarily, the muscle mass of SMID patients with mild malnutrition was 20.67% ± 6.02% of body weight ^(17)^. These results are consistent with our findings.

This study had some limitations. First, the sampling data were obtained from few patients. Our institution only accepts patients with motor and intellectual disabilities. This cohort is relatively smaller than those with either condition; the subgroup of SMID patients that undergo kidney function examination using the clearance methods is also small. However, this cohort are put at risk if eGFR-Cr overestimates their kidney function; thus, evaluation of this measure in such patients albeit a small subgroup is important. Second, eGFR-Ccr ^(4)^ was used as the gold standard for kidney function evaluation instead of Cin. Third, patients with hypoalbuminemia often exhibit increased tubular secretion of creatinine, leading to an overestimation of kidney function ^(18)^. SMID patients are known to induce hypoalbuminemia, and the serum albumin levels in our cases ranged from 3.1 to 4.5 mg/dL and may have partially influenced the overestimation of kidney function by eGFR-Cr.

### Conclusion

We evaluated three eGFR equations, which were designed for Japanese adults and children, in SMID patients. eGFR-Cr is not recommended to be used for the assessment of kidney function in these patients, overestimating by about 1.9 times. It is better to use eGFR-cysC to evaluate kidney function in such patients. As for eGFR-β2MG, its efficacy was close to that of eGFR-cysC, despite the pediatric formula. The kidney function of SMID patients was similar to that of the general population, but their muscle mass was about 60% of that of the general population. The method of measuring muscle mass described in this study can help determine the appropriate weight for SMID patients.

## Article Information

### Conflicts of Interest

None

### Author Contributions

All authors contributed to the study conception and design. Material preparation, data collection, and analysis were performed by OU. The first draft of the manuscript was written by OU. All authors reviewed the previous versions of the manuscript and read and approved the final version.

### Approval by Institutional Review Board (IRB)

The study was approved by the ethics committee of Ichinomiya Medical Treatment & Habilitation Center (approval number: 2021037).

### Informed Consent

Written informed consent was not obtained for use of retrospective clinical data. This information was available on a poster presentation (opt-out).

## References

[1] Uemura O, Nagai T, Ishikura K, et al. Creatinine-based equation to estimate the glomerular filtration rate in Japanese children and adolescents with chronic kidney disease. Clin Exp Nephrol. 2014;18(4):626-33. 24013764 10.1007/s10157-013-0856-y

[2] Uemura O, Nagai T, Ishikura K, et al. Cystatin C-based equation for estimating glomerular filtration rate in Japanese children and adolescents. Clin Exp Nephrol. 2014;18(5):718-25. 24253614 10.1007/s10157-013-0910-9

[3] Ikezumi Y, Uemura O, Nagai T, et al. Beta-2 microglobulin-based equation for estimating glomerular filtration rates in Japanese children and adolescents. Clin Exp Nephrol. 2015;19(3):450-7. 25082657 10.1007/s10157-014-1015-9

[4] Matsuo S, Imai E, Horio M, et al. Revised equations for estimated GFR from serum creatinine in Japan. Am J Kidey Dis. 2009;53(6):982-92. 10.1053/j.ajkd.2008.12.034 19339088

[5] Horio M, Imai E, Yasuda Y, et al. GFR estimation using standardized serum cystatin C in Japan. Am J Kidey Dis. 2013;61(2):197-203. 10.1053/j.ajkd.2012.07.007 22892396

[6] Aldenbratt A, Lindberg C, Johannesson E, et al. Estimation of kidney function in patients with primary neuromuscular diseases: is serum cystatin C a better marker of kidney function than creatinine? J Nephrol. 2022;35(2):493-503. 34351595 10.1007/s40620-021-01122-x PMC8926948

[7] Yoshimura Y, Wakabayashi H, Nagano F, et al. Elevated creatinine-based estimated glomerular filtration rate is associated with increased risk of sarcopenia, dysphagia, and reduced functional recovery after stroke. J Stroke Cerebrovasc Dis. 2021;30(2):105491. 33253988 10.1016/j.jstrokecerebrovasdis.2020.105491

[8] Okita M, Nio K, Murabata M, et al. Reliability and validity of the Japanese version of the Paediatric Pain Profile for children with severe motor and intellectual disabilities. PLoS One. 2020;15(12):e0243566. 33351799 10.1371/journal.pone.0243566 PMC7755203

[9] Nakahara H, Hashizume N, Yoshida M, et al. Creatinine-to-cystatin C ratio estimates muscle mass correlating the markers of the patients with severe motor and intellectual disabilities. Brain Dev. 2022;44(3):196-202. 34782198 10.1016/j.braindev.2021.10.006

[10] Adachi M, Tanaka A, Aiso M, et al. Benefit of cystatin C in evaluation of renal function and prediction of survival in patients with cirrhosis. Hepatol Res. 2015;45(13):1299-306. 25704216 10.1111/hepr.12508

[11] Levey AS, Inker LA, Coresh J. GFR estimation: from physiology to public health. Am J Kidey Dis. 2014;63(5):820-34. 10.1053/j.ajkd.2013.12.006 PMC4001724 24485147

[12] Haines RW, Fowler AJ, Liang K, et al. Comparison of cystatin C and creatinine in the assessment of measured kidney function during critical illness. Clin J Am Soc Nephrol. 2023;18(8):997-1005. 37256861 10.2215/CJN.0000000000000203 PMC10564373

[13] Uemura O, Iwata N, Nagai T, et al. Influence of thyroid function on glomerular filtration rate and other estimates of kidney function in two pediatric patients. CEN Case Reports. 2018;7(1):169-73. 29492844 10.1007/s13730-018-0320-7 PMC5886950

[14] Nagai K, Asahi K, Iseki K, et al. Estimating the prevalence of definitive chronic kidney disease in the Japanese general population. Clin Exp Nephrol. 2021;25(8):885-92. 33839966 10.1007/s10157-021-02049-0

[15] Iseki K, Asahi K, Moriyama T, et al. Risk factor profiles based on estimated glomerular filtration rate and dipstick proteinuria among participants of the Specific Health Check and Guidance System in Japan 2008. Clin Exp Nephrol. 2012;16(2):244-9. 22057582 10.1007/s10157-011-0551-9

[16] Janssen I, Heymsfield SB, Baumgartner RN, et al. Estimation of skeletal muscle mass by bioelectrical impedance analysis. J Appl Physiol. 2000;89(2):465-71. 10926627 10.1152/jappl.2000.89.2.465

[17] Yano N, Iwashita D, Ohwatashi A. The utility of bioelectrical impedance analysis to assess nutritional status of patients with severe motor and intellectual disabilities. Clin Nutr ESPEN. 2022;50:191-5. 35871923 10.1016/j.clnesp.2022.05.018

[18] Horio M, Imai E, Yasuda Y, et al. Lower serum albumin level is associated with higher fractional excretion of creatinine. Clin Exp Nephrol. 2014;18(3):469-74. 23877710 10.1007/s10157-013-0841-5

